# Extremely Non-Equilibrium Hopping Transport and Photogeneration Efficiency in Organic Semiconductors: An Analytic Approach

**DOI:** 10.1021/acs.jpclett.4c00662

**Published:** 2024-04-01

**Authors:** Artem V. Toropin, Libai Huang, Vladimir R. Nikitenko, Oleg V. Prezhdo

**Affiliations:** † Department of Condensed Matter Physics, National Research Nuclear University “MEPhI”, Moscow 115409, Russia; ‡ Department of Chemistry, Purdue University, West Lafayette, Indiana 47907, United States; § Department of Chemistry, University of Southern California, Los Angeles, California 90089, United States

## Abstract

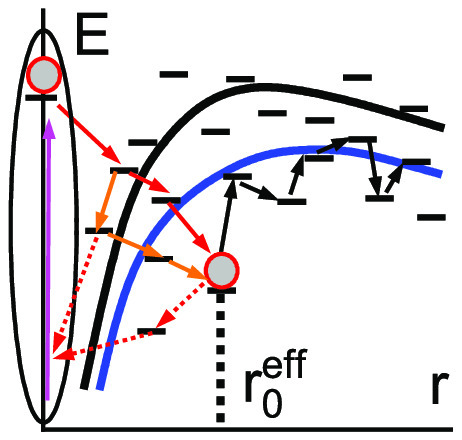

An analytical model of highly nonequilibrium hopping transport of charge carriers in disordered organic semiconductors has been developed. In particular, the initial time interval is considered when transport is controlled by hops down in energy. The model is applied to the calculation of the separation probability of geminate pairs in a semiconductor with a Gaussian energy distribution of localized states. This probability determines the photogeneration efficiency. The temperature dependence of the separation probability is obtained and shown to be much weaker than predicted by the classical Onsager model, in agreement with experiment and Monte Carlo simulations. The field dependence is taken into account using a modified effective temperature method.

Organic semiconductors are already widely used in organic light-emitting diodes, and extensive research continues for their applications in photovoltaics and other organic electronics devices.^1−4^ Despite the enormous diversity of structure and optoelectronic characteristics, organic semiconductors, including both low-molecular materials and polymers, share common transport properties, such as the hopping nature of transport of charge carriers and excitations, as well as structural and associated energy disorder. It is the latter that determines the strong temperature and field dependence of mobility in disordered organic semiconductors, according to the Gaussian disorder model.^5^ Within the framework of this model, many groups have carried out theoretical modeling of the mobility and diffusion coefficient of charge carriers, both by the Monte Carlo method^5−7^ and by analytic methods; see, for example, refs (8−11). It is known that energy disorder leads to long thermalization times for the initially nonequilibrium energy distribution of carriers, for example, in the case of photogeneration. Thermalization occurs during transport, and this leads to anomalous transport characteristics, such as dispersive or extremely nonequilibrium transport.^5,8,12^ However, most studies considered the case of either quasi-equilibrium transport^9−11^ or the case when highly nonequilibrium transport is controlled by thermally activated jumps. In these cases, the drift shift and diffusion spreading of the carriers in a weak field are connected by the Einstein relation.^13^ On the other hand, it has been shown that the Einstein relation does not hold in the limiting case of low temperature, when transport is carried out by jumps down in energy.^14−16^

The mode of jumps down in energy should be taken into account when analyzing the efficiency of photogeneration of charge carriers.^17,18^ There is a strong argument that the primary photoexcitations in organic semiconductors, including conjugated polymers, are molecular excitations (excitons).^19,20^ Exciton decay leads to the formation of electron–hole pairs coupled by Coulomb interaction (geminate pairs). Since the charges (“twins”) must separate by a distance greater than *r*_c_ (Onsager radius, *r*_c_ > 13 nm, at temperature *T* < 300 K) which significantly exceeds the typical hopping length (on the order of a nanometer), the probability of pair separation, which governs the quantum charge photogeneration yield, is determined by the diffusion-drift motion of the “twins” in their Coulomb field and an external uniform electric field. Thus, the separation of geminate pairs is controlled by the transport of charge carriers. Transport can occur in an extremely nonequilibrium regime, especially at early times after a photoexcitation and at low temperatures (thermal energy is much smaller than the scale of energy disorder). However, the effect of energy disorder on the photogeneration of charge carriers has been studied much less than on transport in a uniform electric field. Often theoretical analysis is carried out within the framework of the classical Onsager model, which does not take into account energy disorder and the hopping nature of transport.^21,22^ Monte Carlo simulation^17^ showed that the temperature dependence of mobility in a weak external field at low temperatures does not agree with this model, similarly to the experimental data.^23^ Even if the Onsager model is qualitatively correct (the temperature dependence is described by the Arrhenius law at sufficiently high temperatures), the question arises about the value of the key parameter of the model—the initial distance between the “twins” (initial separation), *r*_0_. Monte Carlo simulations showed that the effective (apparent) magnitude of the initial separation increases with decreasing temperature and is determined by the initial energy relaxation; i.e., downward energy jumps dominate.^17,18^

In a recent work,^18^ we showed, using the model of the exponential energy distribution of hopping centers, that the analytic model of extremely nonequilibrium transport gives a weak temperature dependence in agreement with the Monte Carlo data^17^ and experiment.^23^ In comparison with Monte Carlo, the analytic approach is not associated with specific parameter values and facilitates the analysis of the contribution of various processes to the phenomenon under study. It was shown that anomalously strong diffusion during the initial time interval (after pair generation) leads to an increase in the effective initial separation, which is greater the lower the temperature. However, simplifying assumptions were made in the work.^18^ In particular, the exponential energy distribution of hopping centers was considered, while organic semiconductors are characterized by a Gaussian distribution.

In this work, an analytic model is constructed for an arbitrary (rapidly decreasing in depth, for example, Gaussian) energy distribution of states (DOS), *g*(*E*), and it is shown that the relationship between drift and diffusion in the initial time interval and at low temperatures is determined by the type and width of the DOS, and at large times there is a transition to the Einstein relation. A characteristic time has been found after which the Einstein relation is valid and the Onsager model is applicable. An analytic model of drift and diffusion in an extremely nonequilibrium regime is applied to the problem of the separation probability of a geminate pair. Both temperature and field dependences are analyzed using the effective temperature method modified in this work.

## From the Master Equation to Equations of the Multiple Trapping and Release (MTR) Model.

**Figure 1:**
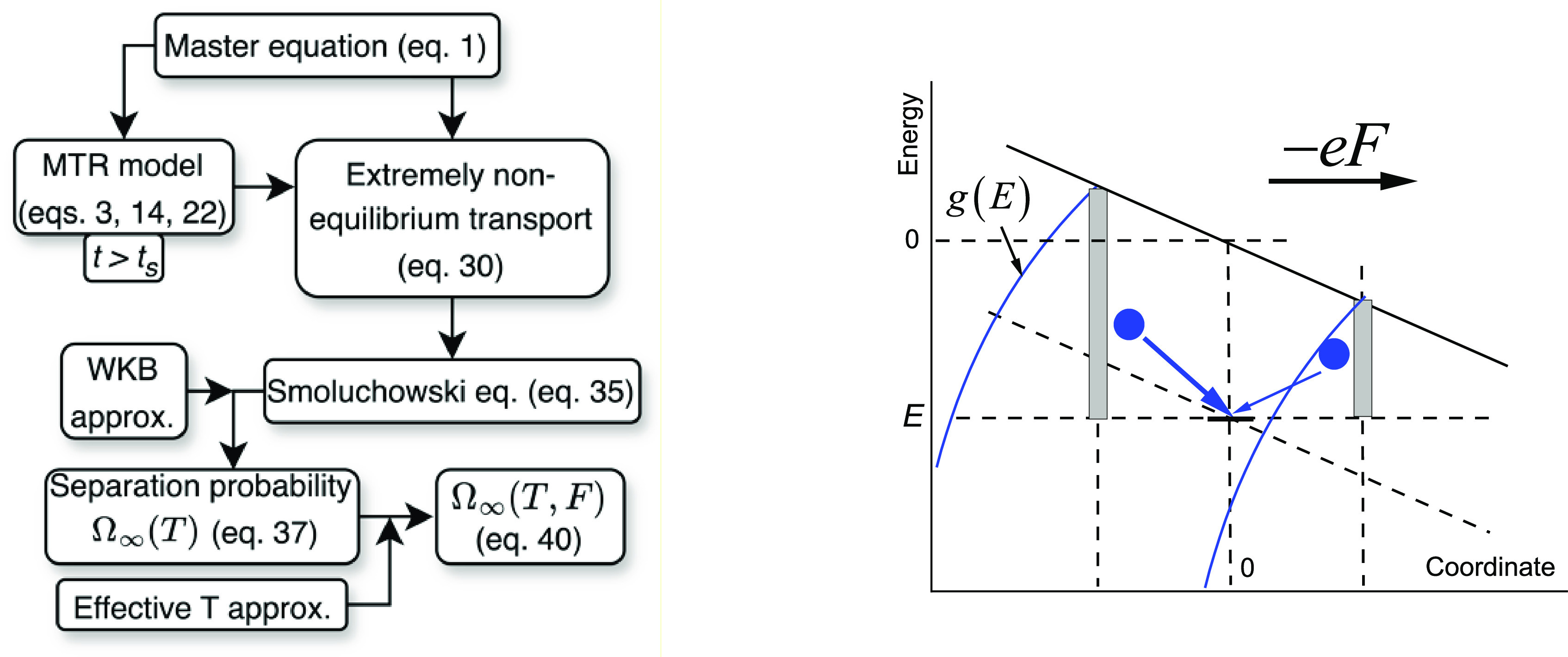
Left: workflow of the derivation. Right: scheme of electron hops in an electric field of strength *F*. The shaded rectangle shows the energy regions of the states from which hops to a state with a given energy *E* are possible at a small temperature.

 The left panel of Figure 1 shows the workflow of the derivation. We start from the well-known master equation for hopping transport^24^
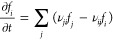
1where *f*_*i*_ ≪ 1 and *f*_*j*_ ≪ 1 are occupation probabilities of states *i* and *j* and ν_*ij*_ and ν_*ji*_ are rates of hopping from *i* to *j* and vice versa, as defined by the Miller–Abraham (MA) model. We describe the localized states by their energy distribution (DOS function), *g*(*E*), and by means of this DOS, we rewrite the equation for the energy distribution of occupied states (ODOS), ρ(*E*, *x*, *t*) = *g*(*E*)*f*(*E*, *x*, *t*), passing from summation to integration
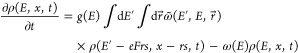
2where *x* is a macroscopic coordinate, *r* is a (microscopic) hopping distance, *e* is the elementary charge, *s* = cos θ, and θ is the angle between the direction of electric force, −*eF⃗*, and the direction of a jump, *r⃗*. Equation 2 takes into account that the electron energy includes the electrostatic energy of the external field; see the right panel of Figure 1.

The concentration of charge carriers (electrons) is
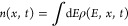
3The rate of escape from a state with energy *E* is
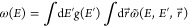
4Unlike the hopping rates ν_*ij*_ = ν(*E*, *E*′, *r*) = ν_0_ exp{−2*γr* – [|*E*_*j*_ – *E*_*i*_| + (*E*_*j*_ – *E*_*i*_)]/2*kT*} in eq 1, the hopping rate ω̃(*E*, *E*′, *r*) takes into account the possibility of going to states other than the final state, as well as the probability that there will be no return to the initial state; see the Supporting Information.

For the small-field limit, one can rewrite eq 4 as
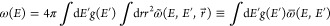
5At first, we consider eq 2 in the zero approximation, assuming *F* = 0 and neglecting the *x*-dependence of the function ρ(*E*, *t*), in order to analyze the energy relaxation itself. Equation 2 reduces to
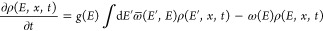
6Considering sufficiently a nonequilibrium (not quasi-equilibrium) process, one has to note that states with a given energy *E* are predominantly filled by carriers from states with energies *E*′, such that the condition ω(*E*′)*t* ≫ 1 is satisfied (i.e., from “currently shallow” states). The time-dependent DOS for these states is *g*_sh_(*E*′, *t*) = *g*(*E*′)[1 – exp[−ω(*E*′)*t*]], according to Poisson’s distribution.^25^ Oppositely, one can neglect transitions from the “currently deep” states; their distribution is *g*_d_(*E*′, *t*) = *g*(*E*′) exp[−ω(*E*′)*t*]. Qualitatively, one can define the “currently shallow” and “currently deep” states by the conditions *E*′ ≥ *E*_d_(*t*) and *E*′ < *E*_d_(*t*), respectively.

The demarcation energy, *E*_d_(*t*), is defined by the condition
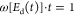
7The detailed balance principle
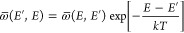
8and expressing the ODOS in a separable form^10,16^ as
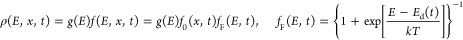
9
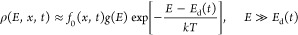
10allow us to modify eq 6 to
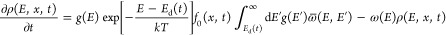
11One can write a nonequilibrium energy distribution, ρ(*E*, *x*, *t*), in the form of eq 9 or 10, considering that (1) the length scale of variations with *x* considerably exceeds the hopping length; (2) ρ(*E*) follows the Boltzmann function, if *E* ≫ *E*_d_(*t*); (3) ρ(*E*) is a constant, if *E* ≪ *E*_d_(*t*), because the rate of downward jumps does not depend on the final energy (Miller–Abrahams model).

The release of a carrier from a state of the energy *E* by jumps downward and upward in energy prevails under the conditions *E* > *E*_1/2_ and *E* < *E*_1/2_, respectively;^13,16,18^ see part S1 of the Supporting Information (SI) about the energy *E*_1/2_. After the “segregation time” *t*_s_, which is defined by the condition *E*_d_(*t*_s_) = *E*_1/2_, transport is controlled by thermally activated jumps, while downward (in energy) jumps prevail earlier.^8,13,26^ Taking the limit *E*_d_(*t*) → −∞ in the integral in eq 11 at *t* ≫ *t*_s_, using eqs 5, 10, and 11 and expressing
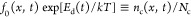
12one obtains
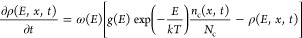
13or
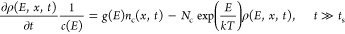
14where *c*(*E*) = ω(*E*) exp(−*E*/*kT*)/*N*_c_. Equation 14 is the balance equation of the MTR model.^27,28^*N*_c_ and *n*_c_(*x*, *t*) are the concentrations of “conductive” states (i.e., the states giving the main contribution to transport) and charge carriers in these states (“mobile carriers”), respectively. Equation 14 has been derived in the work^11^ for the quasi-equilibrium conditions.

Returning to eq 2, we expand its right-hand side in a power series in small parameters *eFrs* and *rs* up to the second order, assuming that only carriers from “shallow” states can contribute to the transport process. Keeping only the first power of electric field (assuming weak field), we obtain after the integration over energy *E*
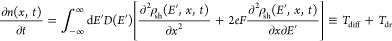
15where
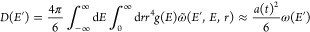
16where *a* is the typical hopping distance; see below.

Using eq 9, one can rewrite the first (diffusion) term in the right-hand side of eq 15 as
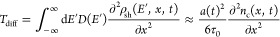
17where
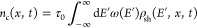
18is the concentration of “mobile” carriers and τ_0_ is the typical escape time from “conductive states”. Indeed, eq 18 follows from the conditions that the escape time from a state with the given energy *E*′ is smaller than τ_0_ and the time τ_0_ is rather small, ω(*E*′)τ_0_ ≪ 1; hence the Poisson’s probability that the state is “conductive” is approximately equal to ω(*E*′)τ_0_: 1 – exp[−ω(*E*′)τ_0_] ≈ ω(*E*′)τ_0_.^11,29^ One has to note that ω(*E*′) ≈ ν_0_ exp[−(*E*_tr_ – *E*′)/*kT*], *E*′ ≪ *E*_1/2_, assuming Miller–Abraham hopping rates, where *E*_tr_ is the effective transport level;^10,13^ see also part S1 in the SI. Hence in eqs 13 and 14
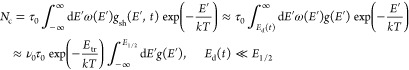
19i.e., it is a constant at *t* ≫ *t*_s_ if the function *g*(*E*′) decreases rapidly with energy *E*′.

Integration in parts in the second (drift) term in eq 15, using eqs 8–10, gives
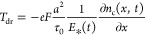
20
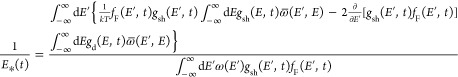
21Thus, one can express eq 15 in the MTR model form
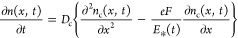
22where *D*_c_ = *a*(*t*)^2^/(6τ_0_) is the coefficient of diffusion of “mobile carriers”. Equations 3, 14, and 22 form a complete set of MTR model equations. Since only a minority of charge carriers can be classified as “mobile” (ω(*E*′)τ_0_ ≪ 1), their concentration is neglected in eq 3. One has to note that the value of τ_0_ disappears from eqs 15 and 22; see eqs 18 and 19.

Strictly speaking, eq 14 is valid in a limited time interval, *t* ≫ *t*_s_; however, one can consider also the initial time interval (*t* ≪ *t*_s_) in the extremely nonequilibrium transport regime, assuming that the majority of carriers occupy “currently deep” states, i.e., *n*(*x*, *t*) ≈ *f*_0_(*x*, *t*)∫d*Eg*_d_(*E*, *t*)*f*_F_(*E*, *t*). For the “currently deep” states, one can neglect the second (release) term in eqs 11 and 14:
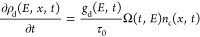
23
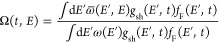
24Integration of eq 23 over energy *E* and then over time gives the relationship known in the theory of extremely nonequilibrium transport,^13,25,30^
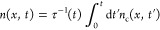
25i.e.,
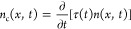
26where
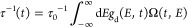
27The difference from the usual expression^13,25,30^
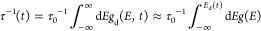
28is only in the appearance of the function Ω(*t*, *E*). In deriving the above equations, the adiabatic approximation was used,^13,25^ according to which the function *n*_c_(*x*, *t*′) changes much faster than the function *g*_d_(*E*, *t*), due to fast hopping between “conductive” states. The approximation of “extremely non-equilibrium transport” is valid under the condition of rather strong disorder, *kT* ≪ *E*_0_, where *E*_0_ is the energy scale of the DOS *g*(*E*), at a limited initial time interval, *t* ≪ *t*_eq_. For example, *E*_0_ = σ, *t*_eq_ is defined by the condition^5^*E*_d_(*t*_eq_) = −σ^2^/*kT* for the Gaussian function *g*(*E*) with the variance σ.

To obtain the equation for the total concentration of charge carriers, *n*(*x*, *t*), we analyze the time dependence of the characteristic energy E_*_(*t*) in the realistic case *kT* ≪ *E*_0_; see eq 21. Replacing ∫_–∞_^∞^d*Eg*_sh_(*E*, *t*)ω̅(*E*′, *E*) = ∫_–∞_^∞^d*E*(*g*(*E*) – *g*_d_(*E*, *t*))ω̅(*E*′, *E*) in the first term of eq 21 and using eqs 5 and 23, one can express this term as (1/*kT*)[1 – τ_0_/τ(*t*)]. In the long-time limit, *t* ≫ *t*_s_, *g*_sh_(*E*, *t*) ≈ *g*(*E*), except for a deep tail. Hence, the first term of eq 21 approaches 1/*kT*, while the second term approaches zero with the concentration of “currently deep” states. Vice versa, the second term of eq 21 prevails at the initial time period, *t* ≪ *t*_s_, and downward jumps from the narrow layer near the demarcation energy *E*_d_(*t*) control the transport.^14,16^ Hence, the typical release rate is ω(*E*_d_) = *t*^–1^. An estimation gives that the first term is small as *kT*/*E*_0_. Equation 9 approaches the step function, and one can estimate *f*_0_(*x*, *t*) ≈ *n*(*x*, *t*)/*G*[*E*_d_(*t*)] in eq 9, where *G*(*E*) ≡ ∫_–∞_^*E*^d*Eg*(*E*). Since downward jumps prevail, i.e., *E*′ > *E*, ∫_–∞_^*E*^′^^d*Eg*(*E*)ω̅(*E*′, *E*) ≈ ω(*E*′) ≈ 1/*t*, *E*′ ≈ *E*_d_(*t*). Estimating the second term in eq 21, multiplied by ∂*n*_c_(*x*, *t*)/∂*x*, and using eq 9, one obtains
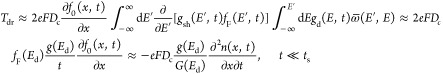
since one can estimate ∂*f*_0_(*x*, *t*)/∂*t* ≈ *f*_0_(*x*, *t*)ω[*E*_d_(*t*)] = *f*_0_(*x*, *t*)/*t*.^16,18^

Combining the above estimations and eqs 22 and 26, one obtains
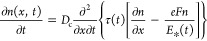
29Integration over time gives
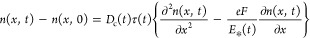
30where *D*_c_(*t*) = *a*^2^(*t*)/(6τ_0_), and the energy E_*_(*t*) is estimated as
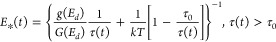
31At the initial time period, *t* ≪ *t*_s_, “conductive states” are located near the demarcation energy *E*_d_(*t*), and the typical hopping length results from the condition ω(*E*_d_) = *t*^–1^ = ν_0_ exp(−2*γa*). Hence, *a*(*t*) = (2γ)^−1^ ln(ν_0_*t*), *t* < *t*_s_. At long times, “conductive states” are located near the transport level *E*_1/2_ = *E*_d_(*t*_s_). Hence, *a* = (2γ)^−1^ ln(ν_0_*t*_s_), *t* ≥ *t*_s_. Only one nearest hopping state exists for the downward jump from the states near the demarcation energy at *t* ≪ *t*_s_ (one can neglect the probability of return), *G*[*E*_d_(*t*)](4π/3)*a*(*t*)^3^ = 1. Hence, one obtains the asymptotic dependence *E*_d_(*t*) from the equation
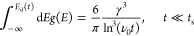
32Since the majority of jumps occur from “currently shallow” to “currently deep” states, ∫d*Eg*_d_(*E*, *t*)ω̅(*E*′, *E*) ≈ ω(*E*′) in eqs 24 and 27, in accordance with eq 5. Hence,
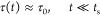
33In the opposite case, *t* ≫ *t*_s_, ω(*E*′) = ν_0_ exp[−(*E*_tr_ – *E*′)/*kT*] for the majority (*E* ≪ *E*_1/2_) of occupied “currently shallow” states,^10,13^ and *f*_F_(*E*, *t*) ≈ exp[−(*E* – *E*_d_)/*kT*], *E*_d_(*t*) ≈ *E*_tr_ – *kT* ln(ν_0_*t*); one can estimate Ω ≈ *G*[*E*_1/2_]^−1^ from eq 24. Hence from eq 27
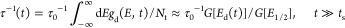
34the same as in the usual MTR approach in the dispersive transport regime, where *N*_t_ = *G*[*E*_1/2_] is the total concentration of “traps”, i. e., states below the transport energy *E*_1/2_, that is an analog of the mobility edge of the MTR model.^13^

## Separation Probability of Geminate Pairs.

 On the basis of the above results, we can write a Smoluchowski equation for the dispersive transport regime of “geminies”^18,31^
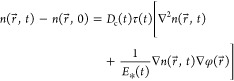
35For the case of a negligible external field (the case of central symmetry, φ(*r*) = −*e*/4πεε_0_*r*) eq 35 takes the form
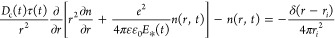
36provided that *n*(*r*, 0) = δ(*r* – *r*_*i*_)/4π*r*_*i*_^2^; *r*_*i*_ is the distance between the “twins” right after the exciton dissociation (“zero jump”).

Solution of this equation, *n*(*r*, *t*), is obtained in part S2 of the SI for the case of a negligible external field using the Wentzel–Kramers–Brillouin (WKB) approximation.

In fact, an initial energy relaxation at *t* < *t*_s_ is what is considered in the conventional Onsager model to be the thermalization of “hot” carriers; just this process results in an initial carrier separation at a distance *r*_0_. Then, at *t* > *t*_s_, one can apply the Onsager model. After the initial relaxation, E_*_(*t*) = *kT* = const, and transport kinetics (dispersive or quasi-equilibrium) does not affect the separation probability,^31^ Ω_∞_. We use the spatial distribution of carriers at time *t*_s_, *n*(*r*_0_, *t*_s_) (see the SI), as the distribution over the true initial separations^18^*r*_0_
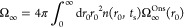
37In eq 37 Ω_∞_^Ons^(*r*_0_) = exp(−*r*_c_/*r*_0_),^21^ where *r*_c_ = *e*^2^/(4πεε_0_*kT*) is the Onsager radius (Coulomb radius) and ε is the relative dielectric permittivity.

## Examples.

**Figure 2:**
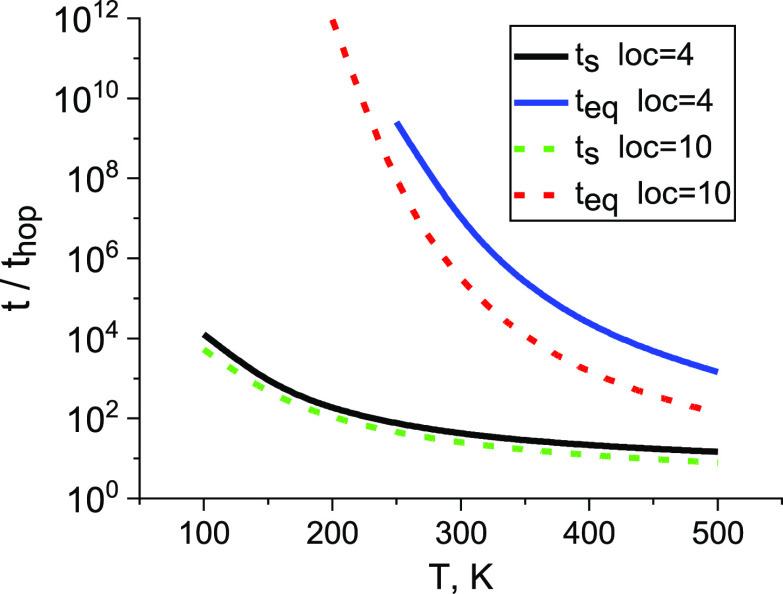
Temperature dependences of the segregation time *t*_s_ and time of equilibration *t*_eq_, both normalized by the hopping time, *t*_hop_ = ν_0_^–1^ exp[−γ(6/π*N*)^1/3^] = 4.94 × 10^–11^ s, loc = 10; *t*_hop_ = 1.2 × 10^–12^ s, loc = 4; ν_0_ = 10^13^ s^–1^. The localization parameter is loc = 2γ*N*^–1/3^.

Equation 30 differs from the respective equation of the conventional MTR model^13,27^ because of the following: (1) time dependences of functions τ(*t*), *D*_c_(*t*) are different at *t* ≪ *t*_s_ (previously these equations were considered only for not too small times, *t* ≫ *t*_s_); (2) the energy E_*_(*t*) in the drift term is time-dependent and differs from the (constant) thermal energy, *kT*. Since τ(*t*) is an increasing function of time, E_*_(*t*) → *kT* in eq 31 in the long-time limit, *t* > *t*_s_, while the first term dominates at short times, *t* ≪ *t*_s_: E_*_(*t*) ≈ *G*(*E*_d_)/*g*(*E*_d_) ≫ *kT*. It is temperature-independent but time-dependent, hence violating the Einstein relation (one has to note, however, that the form of eq 30 differs from the conventional drift-diffusion equation). Equation 30 results from the approximation of extremely nonequilibrium transport; i.e., the majority of carriers occupy “currently deep” states.^25,30^ This approximation is valid at the limited time interval, *t* ≪ *t*_eq_, with *t*_eq_ being the time of quasi-equilibration.^5^ For the case of Gaussian DOS,
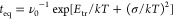
38Both times *t*_s_ and *t*_eq_ increase sharply with decreasing temperature, but the relation *t*_s_ ≪ *t*_eq_ is fulfilled; see Figure 2.

The Einstein relation is fulfilled if the time is rather long, *t* > *t*_s_; i.e., transport is controlled by upward jumps from “currently shallow” (i.e., quasi-equilibrium) states to the states near the transport level *E*_1/2_, that contributes mostly to the transport.^13^ Oppositely, i.e., if *t* ≪ *t*_s_, the thermal energy *kT* is replaced in eqs 30 and 36 by the temperature-independent but time-dependent energy, *G*[*E*_d_(*t*)]/*g*[*E*_d_(*t*)]. For the case of exponential DOS, *G*(*E*_d_)/*g*(*E*_d_) = *E*_0_ = const ≫ *kT*.

**Figure 3:**
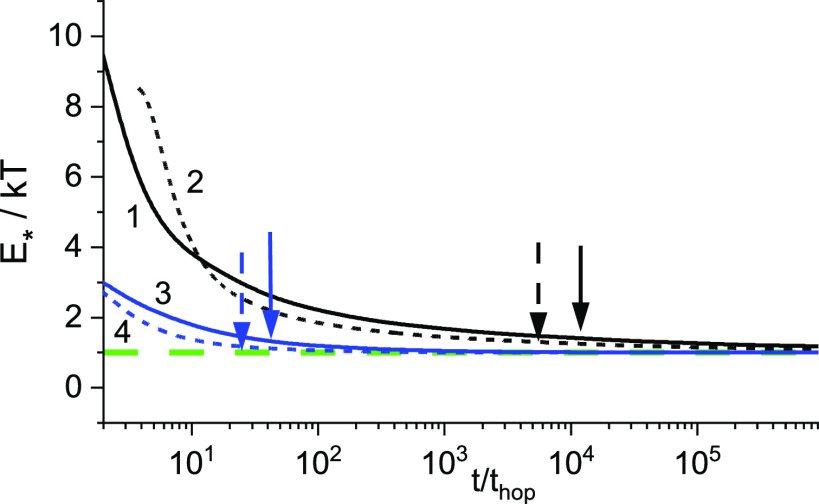
Time dependences of characteristic energy, E_*_(*t*), normalized by thermal energy, *kT*. DOS is Gaussian with σ = 0.1 eV. loc = 10, *t*_hop_ = 4.94 × 10^–11^ s (solid lines). loc = 4, *t*_hop_ = 1.2 × 10^–12^ s (dashed lines). *T* = 100 K (1, 2) and 300 K (3, 4). Arrows show the segregation times, *t*_s_. The horizontal dashed line is the reference level, E_*_ = *kT*.

This circumstance, i.e., the ratio of diffusive dispersion and drift shift scaled by energy *E*_0_ instead of *kT*, was argued previously^14,15^ for the case of exponential DOS, *g*(*E*) = (*N*/*E*_0_) exp[*E*/*E*_0_], *E* < 0, assuming that *T* = 0. The initial time period, *t* ≪ *t*_s_, is similar to the *T* = 0 case in the sense that jumps down in energy predominate. Our analysis shows that, for other (nonexponential) DOS, the energy E_*_ depends on time even at the very initial time interval; see Figure 3.

**Figure 4:**
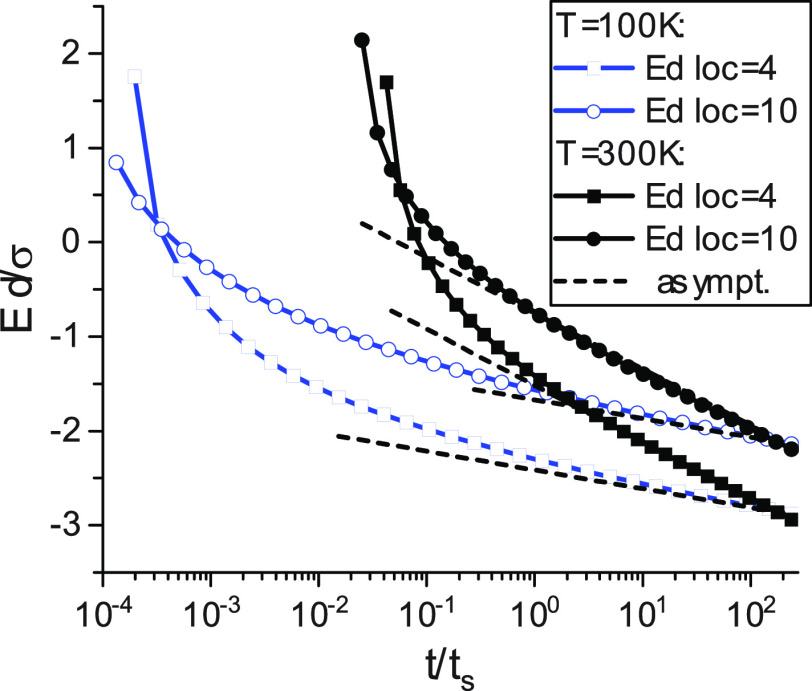
Time dependences of demarcation energy, *E*_d_(*t*), in units of σ. DOS is Gaussian with σ = 0.1 eV. Dashed lines show the long-time asymptote, *E*_d_(*t*) = *E*_tr_ – *kT* ln(ν_0_*t*).

Figure 3 shows that the time interval when E_*_ ≫ *kT* is rather short relative to the segregation time, *t*_s_, although it is long relative to the hopping time, *t*_hop_ = ν_0_^–1^ exp[−γ(6/π*N*)^1/3^], if *T* < 300 K; E_*_ ≈ *kT* at *t* ≥ *t*_s_. The demarcation energy, *E*_d_(*t*), also approaches its asymptotic form, if *t* ≥ *t*_s_; see Figure 4. The duration of the “anomalous” initial time period sharply increases with decreasing temperature.

**Figure 5:**
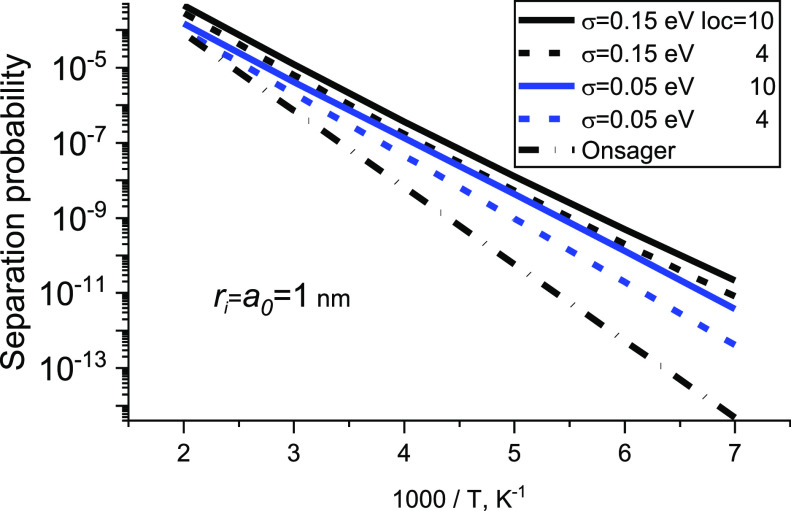
Temperature dependences of separation probability obtained from the model of this work and from the Onsager model, *a*_0_ = *N*^–1/3^.

In the work,^18^ assuming an exponential DOS, the following approximation was used for the function E_*_(*t*) (combination of asymptotes): E_*_(*t*) = *E*_0_, *t* ≤ *t*_s_; E_*_(*t*) = *kT*, *t* > *t*_s_. That is in apparent contradiction with the results of calculations for the Gaussian DOS; see Figure 3. However, calculations of the temperature dependence of separation probability (see eq 37) are in qualitative agreement with the results of the work.^18^ Namely, the separation probability is much larger than the prediction of the Onsager model at low temperatures; see Figure 5.

The separation probability depends on hopping parameters. Namely, it increases with increasing localization parameter, loc = 2*γN*^–1/3^, and with increasing disorder, σ, at a given temperature. One can introduce an effective value of the initial pair separation,^17,18^*r*_0_^ef^. Hence, one can calculate the geminate recombination quantum yield as Ω_∞_(*T*) = exp(−*e*^2^/4πεε_0_*kTr*_0_^ef^(*T*)). This expression does not take into account an external field. Meanwhile, an external field is usually present in both Monte Carlo simulations^17^ and experiments.^23^

The effective temperature concept (ETC), modified in this work, can be used for a joint analysis of the temperature and field dependence of the separation probability. The concept of field-dependent effective temperature *T*_eff_ was proposed initially by Shklovskii,^32^ and it was repeatedly applied later to various disordered materials; see, for example, refs (33 and 34). The essence of ETC is that the electric field *F* reduces the activation energy of jumps by an amount *eFl*, increasing their frequency and, accordingly, mobility. In a disordered medium, in the case of a uniform field, the length scale, *l*, is the localization radius, γ^–1^. An increase in temperature has the same qualitative effect. In the limiting case of *T* = 0 one has *T*_eff_(0, *F*) = λ*eFl*/*k*, where λ is a numerical constant. In general, interpolation expressions such as
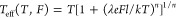
39are used. Usually, *n* = 2, λ = 0.67.^33,34^ The application of the ETC suggests that the quantum yield of geminate recombination can be calculated as
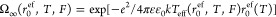
40

The common ETC assumes that the applied field is uniform and release from the initial state is a one-step process. However, in reality, separation of a geminate pair is a multistep process, and the electric field is nonuniform. The carrier undergoes a diffusion–drift motion in the Coulomb potential well. Therefore, one should not expect quantitative results if expression 39 is used in eq 40.

**Figure 6:**
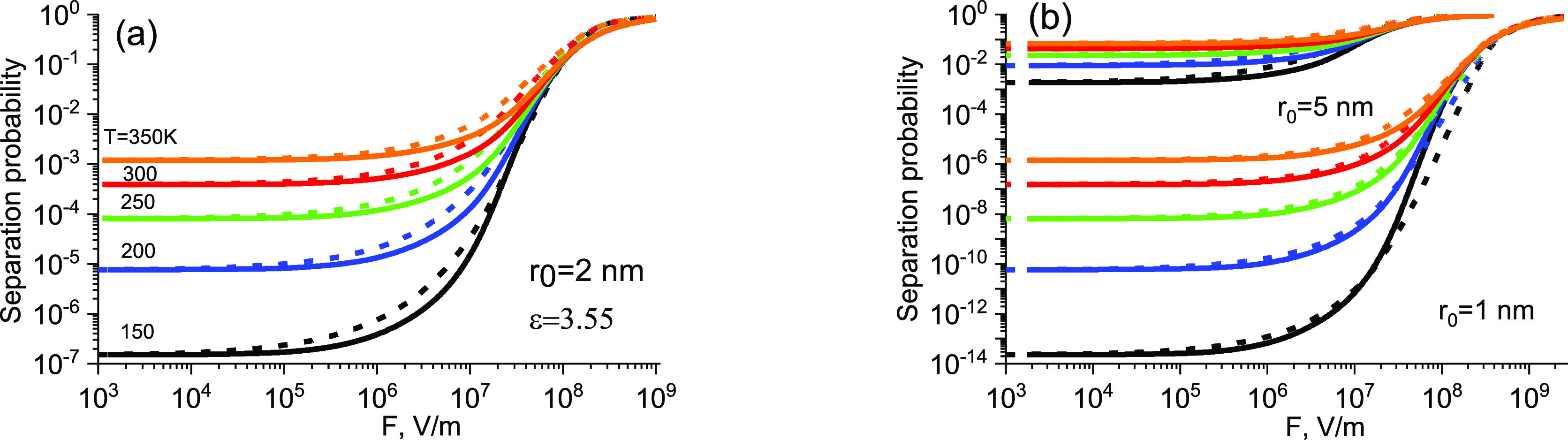
Field and temperature dependences of separation probability, as calculated from the Onsager model (solid lines) and from the modified effective temperature method; see eqs 39–41 (dashed lines). Temperatures in panel (b) are the same as those in panel (a).

**Figure 7:**
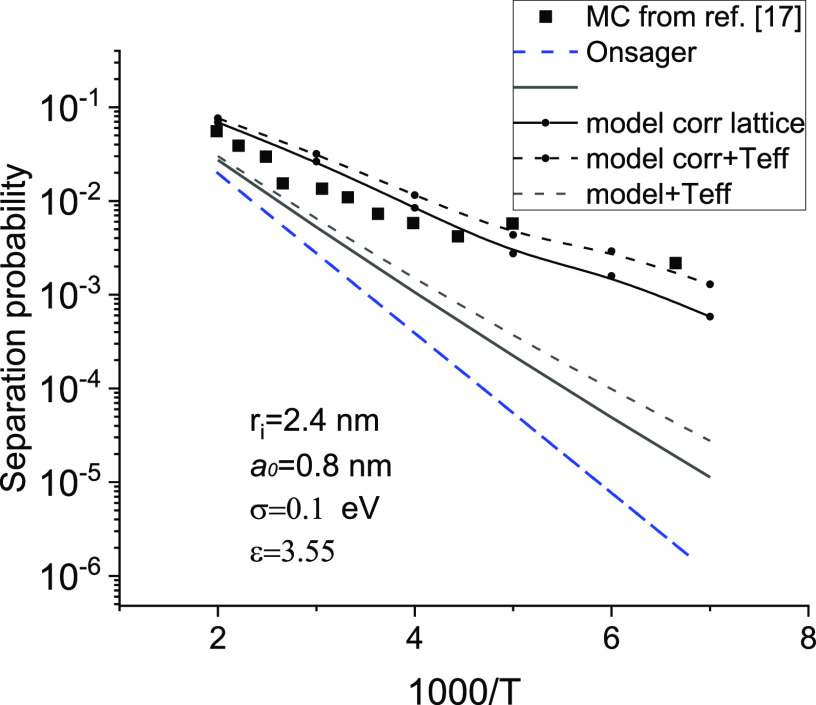
Comparison of the results of this model (solid line) and its modifications (by the use of effective temperature) for the case of regular lattice and finite electric field, *F* = 10^6^ V/m (thin lines), with the results of the Onsager model (dashed line) and the MC modeling^17^ (squares).

At the same time, the ETC provides a qualitatively correct dependence Ω_∞_(*r*_0_, *T*, *F*) in a practically significant interval, 1 nm < *r*_0_^eff^ < 5 nm, and over a fairly wide range of field strength and temperature (see Figure 6) after the following modification. Since in the Onsager model the quantum yield depends on the parameter *eFr*_0_/2*kT*,^21^ the dependence of the exponent *n* in eq 39 on this parameter is assumed. A good fit to the Onsager model is achieved by increasing the value of *n* from 1 to 4 with increasing field *F*. The distance *r*_0_ serves as a spatial scale. Thus, in eq 39
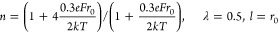
41A similar recent modification of the ETC made it possible to obtain an analytical expression for the temperature and field dependence of mobility in materials with correlated disorder.^35^ An apparently weak field, e.g., 10^6^ V/m, which is insignificant at room temperature, significantly increases the quantum yield at low temperatures; see Figure 6a. One takes this circumstance into account when analyzing the temperature dependence of the quantum yield, obtained experimentally or by MC simulation. The concentration of hopping centers and nondiagonal disorder are also of great importance; see Figure 7. The values obtained with the model developed in this work (solid line) significantly exceed the predictions of the Onsager model (with *r*_0_ = *r*_*i*_ = 2.4 nm at low temperatures) but remain considerably smaller than the MC data^17^ (squares), even after taking into account the uniform field by eqs 39–41 (dashed line). One should note, however, that the MC simulation^17^ used a cubic lattice of hopping centers, while our model assumes a broad spatial distribution of hopping centers, arising due to anisotropy of overlapping wave functions.^5^ As a result, both the typical jump length and the effective initial separation in the case of MC data^17^ should be larger than in our model. Hence, one has to replace *r*_0_^ef^ by *r*_0_^ef^ + Δ*r*_0_ in eq 40. An estimate of the correction to the effective initial separation, Δ*r*_0_, is given in part S3 of the SI. The corrected model results (see the thin lines with small symbols in Figure 7) are in good agreement with the MC data.

**Figure 8:**
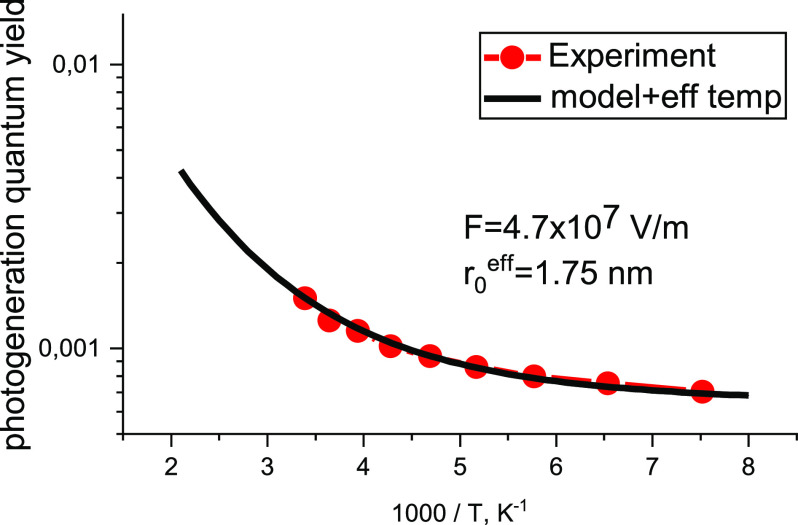
Comparison of the results according to the model of this work (multiplied by the estimated dissociation probability of the primary excitation, 0.18) with the experimental data^23^ for phenyl-substituted polyphenylvinelene (PhPPV) with aluminum electrodes. The field *F* is the same as in the experiment; see caption of Figure 10 in ref (23). loc = 10, σ = 0.1 eV, *r*_*i*_ = 1.4 nm.

The calculations based on the developed model, including the external electric field (see eq 40), also give an excellent agreement with the experimental photogeneration quantum yield, controlled by geminate recombination,^23^Figure 8, providing a realistic value, *r*_0_^ef^ = 1.75 nm. The experimental data are taken from Figure 10 (open squares) of ref (23).

In conclusion, a theoretical model describing the time dependence of the relation between the drift shift and diffusion spreading of the carriers for a realistic Gaussian energy distribution of hopping centers showed that the Einstein relation breaks down over a shorter time interval than that assumed in the previous work.^18^ However, the calculation results qualitatively confirm the conclusion obtained in this work by analytic modeling (and earlier Monte Carlo simulations^17^) that the separation probability of a geminate pair at low temperatures is significantly greater than that predicted by the Onsager model, since an anomalously strong diffusion increases the effective initial separation of the pair. A significant contribution to both the increase in the absolute value of the separation probability and the slowdown of its temperature dependence is also made by the external electric field, which cannot be considered weak at low temperatures and early times with a highly nonequilibrium transport. The results of the calculations using simple analytic formulas (see eqs 37 and 40) and their comparison with the MC simulation data^17^ also stress the effect of off-diagonal disorder, which is opposite to the effect of diagonal (energy) disorder. For a given concentration of hopping centers, the effective initial separation distance and the probability of subsequent separation are smaller, if off-diagonal disorder is stronger.

## Supporting Information Available

Appendices with derivations for the expressions for characteristic time dependences, energies and times (S1), solution for the survival probability in the WKB approximation (S2), and correction of the initial separation to the lattice model (S3) (PDF)

## References

[ref1] Zhang B.; Bonner J. C.; Xu W.; Piper R. T.; Murthy L. N. S.; Hsu J. W. P. Contributions of Charge Generation Versus Transport to Photocurrent in Dilute-Donor Organic Solar Cells with Non-Fullerene Acceptors. J. Phys. Chem. C 2022, 126, 20793–20799. 10.1021/acs.jpcc.2c07821.

[ref2] Deng W.; Liu W.; Qian R.; Wu H. Toward High-Efficiency Organic Photovoltaics: Perspectives on the Origin and Role of Energetic Disorder. J. Phys. Chem. Lett. 2022, 13, 544–551. 10.1021/acs.jpclett.1c03901. 35007067

[ref3] Chen Z.; Zhu H. Photoinduced Charge Transfer and Recombination Dynamics in Star Nonfullerene Organic Solar Cells. J. Phys. Chem. Lett. 2022, 13, 1123–1130. 10.1021/acs.jpclett.1c04247. 35080888

[ref4] Mondal S.; Chowdhury U.; Dey S.; Habib M.; Perez C. M.; Frauenheim T.; Sarkar R.; Pal S.; Prezhdo O. V. Controlling Charge Carrier Dynamics in Porphyrin Nanorings by Optically Active Templates. J. Phys. Chem. Lett. 2023, 14, 11384–11392. 10.1021/acs.jpclett.3c03304. 38078872 PMC10749466

[ref5] Bässler H. Charge Transport in Disordered Organic Photoconductors a Monte Carlo Simulation Study. Phys. Status Solidi B 1993, 175, 15–56. 10.1002/pssb.2221750102.

[ref6] Novikov S. V.; Vannikov A. V. Hopping Charge Transport in Disordered Organic Materials: Where Is the Disorder?. J. Phys. Chem. C 2009, 113, 2532–2540. 10.1021/jp808578b.

[ref7] van der Holst J. J. M.; van Oost F. W. A.; Coehoorn R.; Bobbert P. A. Monte Carlo Study of Charge Transport in Organic Sandwich-Type Single-Carrier Devices: Effects of Coulomb Interactions. Phys. Rev. B 2011, 83, 085206 10.1103/PhysRevB.83.085206.

[ref8] Baranovskii S. D. Mott Lecture: Description of Charge Transport in Disordered Organic Semiconductors: Analytical Theories and Computer Simulations. Phys. Status Solidi 2018, 215, 1700676 10.1002/pssa.201700676.

[ref9] Cottaar J.; Coehoorn R.; Bobbert P. A. Scaling Theory for Percolative Charge Transport in Molecular Semiconductors: Correlated Versus Uncorrelated Energetic Disorder. Phys. Rev. B 2012, 85, 245205 10.1103/PhysRevB.85.245205. 22026880

[ref10] Arkhipov V. I.; Emelianova E. V.; Adriaenssens G. J. Effective Transport Energy Versus the Energy of Most Probable Jumps in Disordered Hopping Systems. Phys. Rev. B 2001, 64, 125125 10.1103/PhysRevB.64.125125.

[ref11] Saxena R.; Nikitenko V. R.; Fishchuk I. I.; Burdakov Ya. V.; Metel Yu. V.; Genoe J.; Bässler H.; Köhler A.; Kadashchuk A. Role of the Reorganization Energy for Charge Transport in Disordered Organic Semiconductors. Phys. Rev. B 2021, 103, 165202 10.1103/PhysRevB.103.165202.

[ref12] Borsenberger P. M.; Bässler H. Tail Broadening of Photocurrent Transients in Molecularly Doped Polymers. J. Appl. Phys. 1994, 75, 967–972. 10.1063/1.356452.

[ref13] Nikitenko V. R.; von Seggern H.; Bässler H. Non-Equilibrium Transport of Charge Carriers in Disordered Organic Materials. J. Phys.: Condens. Matter 2007, 19, 136210 10.1088/0953-8984/19/13/136210.

[ref14] Arkhipov V. I.; Adriaenssens G. J. Low-Temperature Relaxations of Charge Carriers in Disordered Hopping Systems. J. Phys.: Condens. Matter 1996, 8, 7909–7916. 10.1088/0953-8984/8/42/010.

[ref15] Baranovskii S. D.; Faber T.; Hensel F.; Thomas P. On the Einstein Relation for Hopping Electrons. Phys. Stat. Sol. B 1998, 205, 87–90. 10.1002/(SICI)1521-3951(199801)205:1<87::AID-PSSB87>3.0.CO;2-P.

[ref16] Nikitenko V. R. Theoretical Model of Dispersive Tunnel Transport in Disordered Materials. Sov. Phys. Semicond. 1992, 26, 807–811.

[ref17] Albrecht U.; Bässler H. Yield of Geminate Pair Dissociation in an Energetically Random Hopping System. Chem. Phys. Lett. 1995, 235, 389–393. 10.1016/0009-2614(95)00121-J.

[ref18] Toropin A. V.; Nikitenko V. R.; Korolev N. A.; Prezhdo O. V. Disorder and Photogeneration Efficiency in Organic Semiconductors. J. Phys. Chem. Lett. 2023, 14, 7892–7896. 10.1021/acs.jpclett.3c02120. 37639665 PMC10494222

[ref19] Rauscher U.; Bässler H.; Bradley D. D. C.; Hennecke M. Exciton Versus Band Description in the Absorption and Luminescence Spectra in Poly(P-Phenylenevinelene). Phys. Rev. B 1990, 42, 9830–9836. 10.1103/PhysRevB.42.9830. 9995234

[ref20] Bässler H. In Primary Photoexcitations in Conjugated Polymers: Molecular Exciton versus Semiconductor Band Model; Sariciftci N. S., Ed.; World Scientific: Singapore, 1997.

[ref21] Onsager L. Initial Recombination of Ions. Phys. Rev. 1938, 54, 554–557. 10.1103/PhysRev.54.554.

[ref22] Liraz D.; Tessler N. Charge Dissociation in Organic Solar Cells—From Onsager and Frenkel to Modern Models. Chem. Phys. Rev. 2022, 3, 031305 10.1063/5.0099986.

[ref23] Im C.; Emelianova E. V.; Bässler H.; Spreitzer H.; Becker H. Intrinsic and Extrinsic Charge Carrier Photogeneration in Phenyl-Substituted Polyphenylenevinylene-Trinitrofluorenone Blend Systems. J. Chem. Phys. 2002, 117, 2961–2967. 10.1063/1.1490581.

[ref24] Pasveer W. F.; Cottaar J.; Tanase C.; Coehoorn R.; Bobbert P. A.; Blom P. W. M.; de Leeuw D. M.; Michels M. A. J. Unified Description of Charge-Carrier Mobilities in Disordered Semiconducting Polymers. Phys. Rev. Lett. 2005, 94, 206601 10.1103/PhysRevLett.94.206601. 16090265

[ref25] Arkhipov V. I.; Bässler H. An Adiabatic Model of Dispersive Hopping Transport. I. General Results for Weak-Field Drift and Diffusion. Philos. Mag. B 1993, 68, 425–435. 10.1080/13642819308217925.

[ref26] Monroe D. Hopping in Exponential Band Tails. Phys. Rev. Lett. 1985, 54, 146–149. 10.1103/PhysRevLett.54.146. 10031266

[ref27] Rudenko A. I.; Arkhipov V. I. Drift and Diffusion in Materials with Traps. I. Quasi-Equilibrium Transport Regime. Philos. Mag. 1982, 45, 177–187. 10.1080/13642818208246326.

[ref28] Schmidlin F. W. Theory of Trap-Controlled Transient Photoconduction. Phys. Rev. B 1977, 16, 2362–2385. 10.1103/PhysRevB.16.2362.

[ref29] Burdakov Ya. V.; Saunina Yu.; Bässler H.; Köhler A.; Nikitenko V. R. Modeling of Charge Transport in Polymers with Imbedded Crystallites. Phys. Rev. B 2023, 108, 085301 10.1103/PhysRevB.108.085301.

[ref30] Arkhipov V. I.; Rudenko A. I. Drift and Diffusion in Materials with Traps. II. Non-Equilibrium Transport Regime. Philos. Mag. B 1982, 45, 189–207. 10.1080/13642818208246327.

[ref31] Arkhipov V. I.; Nikitenko V. R.; Rudenko A. I.; Shutov S. D. Geminate Recombination Kinetics in Amorphous Semiconductors. J. Non-Cryst. Sol. 1987, 90, 53–56. 10.1016/S0022-3093(87)80382-4.

[ref32] Marianer S.; Shklovskii B. I. Effective Temperature of Hopping Electrons in a Strong Electric Field. Phys. Rev. B 1992, 46, 13100–13103. 10.1103/PhysRevB.46.13100. 10003349

[ref33] Jansson F.; Baranovskii S. D.; Gebhard F.; Österbacka R. Effective Temperature for Hopping Transport in a Gaussian Density of States. Phys. Rev. B 2008, 77, 195211 10.1103/PhysRevB.77.195211.

[ref34] Khan M. D.; Nikitenko V. R.; Tyutnev A. P.; Ikhsanov R. S. Joint Application of Transport Level and Effective Temperature Concepts for an Analytic Description of the Quasi- and Nonequilibrium Charge Transport in Disordered Organics. J. Phys. Chem. C 2019, 123, 1653–1659. 10.1021/acs.jpcc.8b11520.

[ref35] Saunina A. Yu.; Huang L.; Nikitenko V. R.; Prezhdo O. V. On Analytical Modeling of Hopping Transport of Charge Carriers and Excitations in Materials with Correlated Disorder. J. Phys. Chem. Lett. 2024, 15, 2601–2605. 10.1021/acs.jpclett.4c00097. 38416805 PMC10926151

