# Supplementary material for: Extremely Non-Equilibrium Hopping Transport and Photogeneration Efficiency in Organic Semiconductors: An Analytic Approach
Source: J Phys Chem Lett. 2024 Apr 1;15(14):3884–92. doi: 10.1021/acs.jpclett.4c00662 (PMC11017315; doi:10.1021/acs.jpclett.4c00662)
Supplement: Supplementary file 1 — jz4c00662_si_001.pdf [file jz4c00662_si_001.pdf]

Supporting Information for:

Extremely Non-Equilibrium Hopping Transport and Photogeneration Efficiency in Organic Semiconductors: an Analytic Approach

Artem V. Toropin[a], Libai Huang[b], Vladimir R. Nikitenko[a] and Oleg V. Prezhdo[c]

[a] Department of Condensed Matter Physics, National Research Nuclear University "MEPhI", Moscow 115409, Russia

[b]Department of Chemistry, Purdue University, West Lafayette, IN 47907, USA

[c]Department of Chemistry, University of Southern California, Los Angeles, CA 90089, United States

S1. Time dependences, energies and times

One can find the escape rate $\omega(E) \equiv \nu_0 exp\left(-u(E)\right)$ from the following equation [S1],

$$1 = 4\pi \int_{-\infty}^{E+kTu} dE' g(E') \int_0^{r_*(E,E')} dr r^2 W_{esc}(E,E',r)$$

$$\approx \overline{W_{esc}}(E) \frac{4\pi}{3} \int_{-\infty}^{E+kTu} dE' g(E') {r_*}^3(E,E'). \quad \text{(S1)}$$

According to the MA model, the hopping distance is

$$r_*(E,E') = (2\gamma)^{-1}\left[u(E) - \frac{E\prime - E}{kT}\eta(E'-E)\right], \quad \text{(S2)}$$

where $\eta(E'-E)$ is a step function, and $W_{esc}(E,E',r)$ is the probability that the carrier does not return to the initial state after the jump from the state of energy E to the state with energy E' over the distance r. The region of integration in equation S1, i. e., an area in the *r-E* space defined by the condition that the transition rate to any state of this area from the initial state (energy *E*) is not less than a given value ω(*E*), shown in Figure S1. Operationally, we estimate the mean number of available states, $B(E) \equiv \overline{W_{esc}}^{-1}(E)$, from the following interpolation: $B(E) = (n_\downarrow \cdot 1 + n_\uparrow B_0)/(n_\downarrow + n_\uparrow)$, where $n_\uparrow$ and $n_\downarrow$

are the numbers of available neighbor states upwards and downwards in energy for the given escape rate, $\omega(E)$; $B_0 \approx 2.77$.[S2] Since $n_\downarrow(E) + n_\uparrow(E) = B(E)$,

$$B(E) = \sqrt{n_\downarrow(E) + n_\uparrow(E) B_0}, \tag{S3}$$

$$n_\downarrow(E) = \frac{4\pi}{3}\left(\frac{u(E)}{2\gamma}\right)^3 \int_{-\infty}^{E} dE' g(E') = \frac{\pi}{6}\left(\frac{u(E)}{\gamma}\right)^3 G(E), \tag{S4}$$

$$n_\uparrow(E) = \frac{4\pi}{3}\int_{E}^{E+kTu(E)} dE' g(E')\left(\frac{E+kTu(E)-E'}{2\gamma kT}\right)^3 \tag{S5}$$

One can write Eq. S1 as

$$\frac{4\pi}{3}\int_{-\infty}^{E+kTu(E)} dE' g(E') r_*^3(E,E') = B(E) \tag{S6}$$

The value of $B(E)$ approaches unity, if downward jumps prevail, $E \gg E_{1/2,}$ $n_\downarrow(E) \approx 1,$ $n_\uparrow(E) \approx 0$ and one can neglect the possibility of return to the initial state, the same as at zero temperature. Otherwise, transport is controlled by thermally activated jumps to the transport level, and $B(E) \approx B_0$, as was considered previously [S2-S4]. Equations S2-S6 generalize the approach of the work [S3] for calculation of the $\omega(E)$ to the case of short times, $t \ll t_s$.

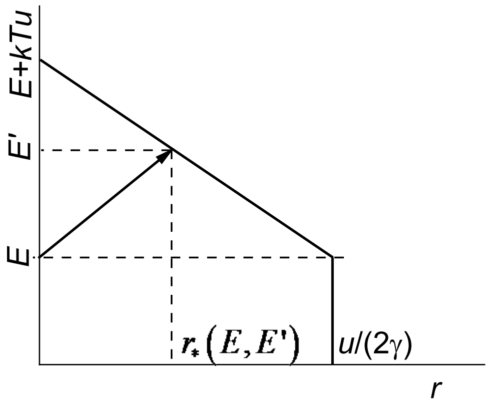

Figure S1. Region in the *r-E'* space containing states for which the condition $\nu(E,E',r) > \omega(E) = \nu_0 exp(-u)$ is fulfilled.

Assuming a sufficiently deep initial state, $E \to -\infty$, neglecting downward jumps for this case, and introducing the energy $E_{tr} = E + kTu$, one can reduce Eq. A6 to the known equation [S4].

$$\frac{4\pi}{3}\int_{-\infty}^{E_{tr}} dE'\, g(E')\left(\frac{E_{tr}-E'}{2\gamma kT}\right)^3 = B_0. \quad \text{(S7)}$$

The energy E_{tr} does not depend on the initial energy E. Hence, the rate of escape from the deep tail of the DOS is

$$\omega = \nu_0 \exp\left(-\frac{E_{tr}-E}{kT}\right), \quad \text{(S8)}$$

where E_{tr} is an *effective* (formal) transport energy [S1, S4].

Since the jumps occur preferably over the distance $r = r_*$, see Fig. S1, one can consider the hopping rate $\tilde{\omega}(E, E', r)$ as

$$\tilde{\omega}(E', E, r) = \omega(E') W_{esc}(E', E, r)\eta(r_*(E', E) - r), \quad \text{(S9)}$$

and $\bar{\omega}(E', E) = \frac{4\pi}{3}\omega(E')W_{esc}(E', E, r)r_*^3(E', E)$ according to Eq. 5 of the main text.

From Eqs. S1-S3 one obtains $G(E)\frac{4\pi}{3}r_*(E)^3 = 1$, $r_*(E) = [(4\pi/3)G(E)]^{-1/3}$, $\omega(E) = \nu_0 exp\big(-2\gamma r_*(E)\big)$, $r_*(t) \equiv r_*[E_d(t)] = (2\gamma)^{-1}ln(\nu_0 t)$, $t \ll t_s$. Since the jumps to the "currently deep" states occur typically downwards in energy, $r_*(E', E) = r_*(E') = (2\gamma)^{-1}ln[\nu_0/\omega(E')]$, one can estimate the functions $\Omega(t, E)$ and $\tau(t)$ in Eqs. 24 and 27 of the main text as

$$\tau^{-1}(t) \approx \tau_0^{-1}\Omega(t)\int_{-\infty}^{\infty} dE\, g_d(E, t), \quad \text{(S10)}$$

$$\Omega(t, E) \approx \Omega(t) \approx \frac{\frac{4\pi}{3}\int_{E_d(t)}^{\infty} dE' g(E')\omega(E')B^{-1}(E')r_*^3(E')\left[1+exp\left[\frac{E'-E_d(t)}{kT}\right]\right]^{-1}}{\int_{E_d(t)}^{\infty} dE' g(E')\omega(E')\left[1+exp\left[\frac{E'-E_d(t)}{kT}\right]\right]^{-1}}, \quad \text{(S11)}$$

The energy $E_{1/2}$ is defined by the condition

$$n_\uparrow(E_{1/2}) = n_\downarrow(E_{1/2}), \quad \text{(S12)}$$

hence, $n_\downarrow(E_{1/2}) = B(E_{1/2})/2$. From Eqs. S3, S12 one obtains

$$B(E_{1/2}) = (1 + B_0)/2. \quad \text{(S13)}$$

One can express the release rate as $\omega(E) = \nu_0 exp[-(E_t(E) - E)/kT]$, $E_{tr} = \lim_{E\to-\infty} E_t(E)$. Eqs. S4-S6, S13 give the equation for the energy $E_{1/2}$,

$$\frac{4\pi}{3}\int_{E_{1/2}}^{E_t(E_{1/2})} dE'\, g(E')\left(\frac{E_t(E_{1/2})-E'}{2\gamma kT}\right)^3 = (1+B_0)/4, \tag{S14}$$

where

$$E_t(E_{1/2}) = E_{1/2} + 2\gamma kT p_0 G^{-1/3}(E_{1/2}),\, p_0 = [3\,(1+B_0)/16\pi]^{1/3}. \tag{S15}$$

Then, one can find the segregation time $t_s = \omega^{-1}(E_{1/2}) = \nu_0^{-1} exp(u_s)$ from the equation

$$u_s = 2\gamma p_0 G^{-1/3}(E_{1/2}), \tag{S16}$$

which follows from Eqs. S4 and S13. One has to note that the energy $E_{1/2}$ plays the role of a *real* (not *effective*) transport level, i. e., the energy, mostly contributing to transport, if $t \gg t_s$.[S5]

S2. Solution for the survival probability in the WKB approximation.

Eq. 36 of the main text is an ordinary differential equation; it includes time as a parameter. Introducing dimensionless variables and parameters, $\tilde{n} = n r_c^{\,3}$, $x = r/r_c$, $x_i = r_i/r_c$, $r_c = e^2/4\pi\varepsilon\varepsilon_0 kT$ (the Coulomb radius), $O^2(t) = r_c^{\,2}/D_0\tau(t)$, $\xi(t) = E_*(t)/kT$, we can rewrite Eq. 36 as

$$\frac{d^2\tilde{n}}{dx^2} + \frac{d\tilde{n}}{dx}\left(\frac{2}{x} + \frac{1}{\xi(t)x^2}\right) - O^2(t)\tilde{n}(r,t) = -O^2(t)\frac{\delta(x-x_i)}{4\pi x_i^{\,2}}. \quad .. \tag{S17}$$

Replacing the function in Eq. S17,

$$\tilde{n} = \frac{O^2(t)}{4\pi x_i x}\exp\left[\frac{1}{2\xi x} - \frac{1}{2\xi x_i}\right] z(x,t), \tag{S18}$$

one obtains the following equations

$$\frac{d^2 z}{dx^2} - \kappa^2(x,t) z(x,t) = -\delta(x-x_i), \tag{S19}$$

$$\kappa(x,t) = \left[\frac{1}{4\xi^2 x^4} + O^2(t)\right]^{1/2}. \tag{S20}$$

Integrating Eq. S19 over an infinitely thin interval around x_i, one obtains the boundary conditions

$$z(x_i+0,t) = z(x_i-0,t), \quad \frac{dz(x_i+0,t)}{dx} - \frac{dz(x_i-0,t)}{dx} = -1. \tag{S21}$$

Other boundary conditions result from the requirement that function $\tilde{n}$ is finite at x=0 and approaches zero as x approaches infinity.

We find the solution of Eq. S19 in the WKB approximation with the boundary conditions S21, and using Eq. S18 obtain

$$n\,(x,t) = r_c^{-3}\frac{O^2(t)\exp\left(\frac{1}{2\xi x}-\frac{1}{2\xi x_i}\right)}{8\pi x_i x\sqrt{k(x,t)k(x_i,t)}}\begin{cases}\exp\left(-\int_x^{x_i}\kappa(x',t)dx'\right), x \le x_i\\ \exp\left(-\int_{x_i}^{x}\kappa(x',t)dx'\right), x > x_i\end{cases} \quad \text{(S22)}$$

We can find integrals in Eq. S22 in terms of hypergeometric functions,

$$\int_x^{x_i} k(x',t)dx' = \frac{{}_2F_1\left(-\frac{1}{2};-\frac{1}{4};\frac{3}{4};-4x^4\xi^2O^2(\mathrm{t})\right)x_i - {}_2F_1\left(-\frac{1}{2};-\frac{1}{4};\frac{3}{4};-4x_i^4\xi^2O^2(\mathrm{t})\right)x}{2xx_i\xi}. \quad \text{(S23)}$$

Calculations show that the applicability condition of the WKB approximation, i. e. $O(t) \gg 1$, is satisfied up to time $t = t_s$ for all parameters used.

S3. Correction of initial separation to the lattice model.

To estimate the correction to the effective initial separation of the geminate pair, ${r_0}^{ef}$, we will approximately assume that only jumps down in energy occur during the initial time period, $t < t_S$. Since the hopping rate does not depend on the final state energy, the number of accessible (i. e., lower in energy) states after each jump is reduced by a factor of two on average [S6]. Thus, the lengths of the m+1st and mth jumps are related as $r_{m+1} = 2^{1/3}r_m$, hence the diffusion length is

$$R_M = r_1\sqrt{\sum_{m=0}^{M} 2^{2m/3}}, \quad \text{(S24)}$$

where M is the number of jumps after which the carrier drops below the level $E_{1/2}$, which is determined by the condition

$$N/2^{M-1} = \int_{-\infty}^{E_{1/2}} dEg(E) = G\left(E_{1/2}\right) \quad \text{(S25)}$$

as an integer part, i. e.,

$$M = \left[1 - log_2\left(G\left(E_{1/2}\right)/N\right)\right]. \quad \text{(S26)}$$

For the case of a simple cubic lattice with the constant a_0, $r_1 = a_0 = N^{-1/3}$, while assuming the off-diagonal disorder we have $r_1 = (4\pi N/3)^{-1/3}$. Thus, one can estimate the correction to the effective initial separation for the comparison with the lattice model as

$$\Delta r_0 = N^{-1/3}\left[1 - (4\pi/3)^{-1/3}\right]\sqrt{\sum_{m=0}^{M} 2^{2m/3}}. \quad \text{(S27)}$$

AUTHOR INFORMATION

Corresponding Authors

* E-mail: vladronik@yandex.ru V.R. Nikitenko, prezhdo@usc.edu O.V. Prezhdo

References

(S1) Arkhipov, V. I.; Emelianova, E. V.; Adriaenssens, G. J. Effective Transport Energy Versus the Energy of Most Probable Jumps in Disordered Hopping Systems. *Phys. Rev. B* **2001**, *64*, 125125.

(S2) Baranovskii, S. D. Mott Lecture: Description of Charge Transport in Disordered Organic Semiconductors: Analytical Theories and Computer Simulations. *Phys. Status Solidi* **2018**, *215*, 1700676.

(S3) Burdakov, Ya. V.; Saunina, A. Yu.; Bässler, H.; Köhler, A.; Nikitenko, V. R. Modeling of Charge Transport in Polymers with Imbedded Crystallites. *Phys. Rev. B* **2023**, *108*, 085301.

(S4) Khan, M. D.; Nikitenko, V. R.; Tyutnev, A. P.; Ikhsanov, R. S. Joint Application of Transport Level and Effective Temperature Concepts for an Analytic Description of the Quasi- and Nonequilibrium Charge Transport in Disordered Organics. *J. Phys. Chem. C* **2019**, *123*, 1653–1659.

(S5) Nikitenko, V. R.; Seggern, H. von; Bässler, H. Non-Equilibrium Transport of Charge Carriers in Disordered Organic Materials. *J. Phys. Condens. Matter* **2007**, *19*, 136210.

(S6) Baranovskii, S. D.; Fritzsche, H.; Levin, E. I.; Ruzin, I. M.; Shklovskii, B. I. Theory of Low-Temperature Photoconductivity and Photoluminescence in Amorphous Semiconductors. *Zh. Eksp. Teor. Fiz*. **1989**, *96*, 1362–1380.
